# Co(OH)_2_ Nanosheets Supported on Laser Ablated Cu Foam: An Efficient Oxygen Evolution Reaction Electrocatalyst

**DOI:** 10.3389/fchem.2019.00900

**Published:** 2020-01-10

**Authors:** Xinfeng Zhou, Weihong Qi, Kai Yin, Ning Zhang, Shen Gong, Zhou Li, Yejun Li

**Affiliations:** ^1^School of Materials Science and Engineering, Central South University, Changsha, China; ^2^State Key Laboratory of Solidification Processing, Center of Advanced Lubrication and Seal Materials, Northwestern Polytechnical University, Xi'an, China; ^3^Hunan Key Laboratory of Super Microstructure and Ultrafast Process, School of Physics and Electronics, Central South University, Changsha, China

**Keywords:** laser fabrication, electrocatalysis, sustainable chemistry, transition metal oxide, water splitting

## Abstract

Highly efficient and low-cost non-noble metal based electrocatalysts for oxygen evolution reaction (OER) have attracted more and more attention in recent years. However, the current research has been focused on the construction of novel OER electrocatalysts themselves, little attention has been paid to the modification of the substrates. In this work, a different strategy is proposed via laser ablation to fabricate the Cu foams with rich micro/nano-structures as OER substrates. Later, the precipitation conversion method was utilized to grow cobalt hydroxide on the laser fabricated Cu foams. The as-produced Cu/Cu oxides/Co(OH)_2_ electrocatalysts exhibit high OER activity in 1 M KOH, requiring an overpotential of only 259 mV at a current density of 50 mA cm^−2^ with excellent mild-term durability. The improved catalytic performance of the prepared samples can be attributed to the increased surface area, rich active sites, and the superhydrophilicity of the laser produced micro/nano-structures.

**Graphical Abstract:**
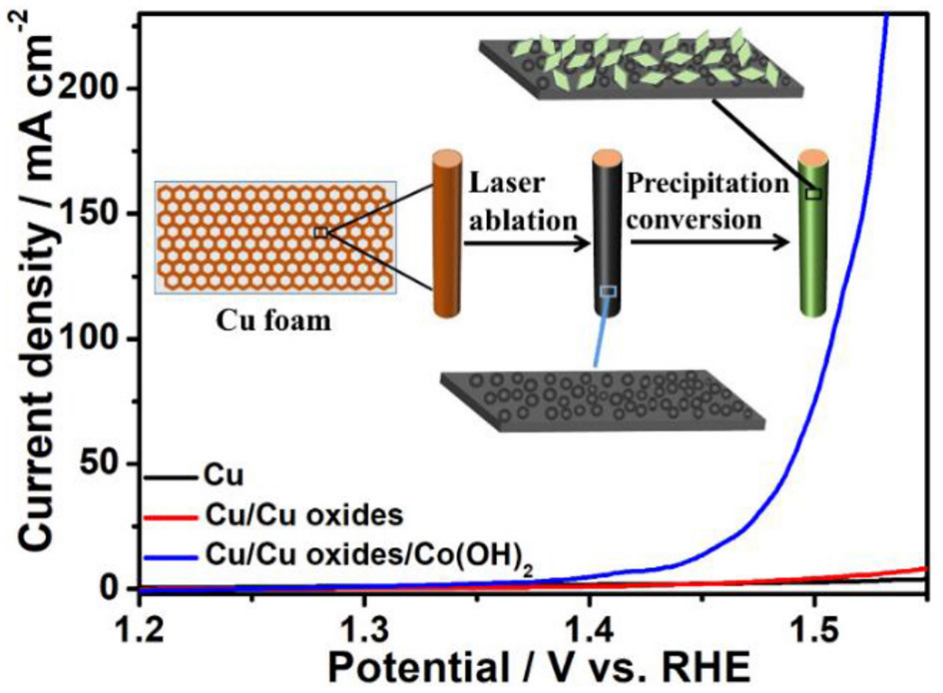
Herein, laser fabrication technique was utilized to modify the solid state surface for the construction and design of active water oxidation catalysts, where the fabricated Cu/Cu oxides/Co(OH)_2_ electrocatalysts show great OER activity and durability in alkaline condition. The present work suggests a new strategy to prepare OER electrocatalysts via substrate-modification.

## Introduction

As a high-power density and zero carbon emission renewable energy carrier, hydrogen (H_2_) has been considered as an ideal alternative energy of fossil fuels (Li et al., 2016, 2019a; Shi and Zhang, 2016; Jin J. et al., 2017; Meng et al., 2017). Electrochemical water splitting has been suggested as an effective approach to produce hydrogen, which has attracted tremendous interests. However, the efficiency of the electrochemical water splitting is greatly hindered by oxygen evolution reaction (OER), which contains a complicated progress of 4H^+^/4e^−^ transfer with sluggish reaction kinetics (Gong et al., 2013; Lu et al., 2016; Ma et al., 2016; Xia et al., 2016; Wu et al., 2017; Lv et al., 2019). Therefore, water oxidation catalysts are needed to lower the OER overpotential, such as noble metal-based catalysts (ruthenium and iridium), which, however, are restricted from widespread industrial application due to the limited supply and high price (Lee et al., 2012; Ai et al., 2017). To satisfy the industrial demand, it is of great importance to develop high efficient OER electrocatalysts based on non-noble metals in the future.

In recent years, transition metals, such as copper and nickel foams, have been widely used as substrates of electrocatalysts for efficient water splitting, due to the low cost, high conductivity, and large surface area. For example, Xiong et al. reported the Co-doped CuO nanoarrays on Cu foam as the efficient and stable anode for electrochemical water splitting, with an overpotential of 299 mV at current densities of 50 mA cm^−2^ (Xiong et al., 2018). A 3D OER electrocatalyst with Cu(OH)_2_@CoCO_3_(OH)_2_·*n*H_2_O heterostructures on Cu foam has been developed by Xie et al. (2017), exhibiting high OER activity with only 290 mV to achieve 100 mA cm^−2^ in 1 M KOH. Hierarchical NiCo_2_S_4_ nanowire arrays have been grown on Ni foam as bifunctional electrocatalysts for both oxygen and hydrogen evolution reactions, which shows stable catalytic activity with overpotentials of 260 mV and 210 mV for OER and HER in 1 M KOH, respectively (Sivanantham et al., 2016).

Despite of these efforts, the current research has been focused on the construction of novel OER electrocatalysts themselves, while little attention has been paid to the modification of the substrates. Laser fabrication has been suggested as an effective approach to modify the solid surface with rich micro/nano-structures, large surface area, and rich defects, as well as superhydrophilicity (Yin et al., 2017a; Yang et al., 2018; Li et al., 2019b; Wu et al., 2019). For Cu foams, the produced Cu oxides have been found to be active in OER (Li et al., 2019b). Moreover, the modified Cu surface with rich micro/nano-structures can be beneficial to the growth of the electrocatalysts as well as to the release of the produced O_2_ gas, thus increase the active sites as well as the catalytic activity. Furthermore, the laser ablation can be a surface fabrication process by adjusting the laser parameters, where the metallic Cu core can be largely retained as the conductive substrate. It is therefore of great interests to combine the laser modified substrates with the electrocatalysts to promote the OER process.

In this contribution, femtosecond laser ablation was utilized to fabricate rich micro/nano-structures on Cu foam, while cobalt hydroxide was grown on the fabricated Cu foam via the precipitation conversion method. The as-prepared electrocatalysts exhibited remarkable OER activity and excellent mild-term stability.

## Experimental Section

### Materials and Chemicals

All chemicals and metal salts including potassium hydroxide (KOH), Sodium dodecyl sulfate (SDS), carbamide (CO(NH_2_)_2_), and cobalt chloride (CoCl_2_·6(H_2_O)) were purchased from Aladdin Co., Ltd., and used without further purification.

### Fabrication of Cu Oxides Micro/Nanostructures on Cu Foam

The copper foam was purchased from Kunshan Metal Material Tech., Suzhou, China. The typical line-by-line femtosecond laser irradiation procedure is applied to modify the Cu foam, where detailed information can be found in previous work (Yin et al., 2017b; Duan et al., 2018). In particular, a high-repetition femtosecond laser system (PHAROS, LIGHT CONVERSION, Lithuania) that generates 250 fs pulses at a repetition rate of 75 kHz with a central wavelength of 1,030 nm is employed to fabricate micro/nano structures on the Cu foam surface. The spacing between two adjacent lines is set to be 10 μm. The laser power and speed were set at 8 W and 3 m/s (about 4 s), respectively. After ablation, the samples were carefully cleaned with distilled water.

### Preparation of the Cu/Cu Oxides/Co(OH)_2_

The Cu/Cu oxides/Co(OH)_2_ electrodes were prepared via the precipitation conversion method (Jin H. et al., 2017). In a typical procedure, a 0.23 g mixture of CoCl_2_ · 6H_2_O (40 mg), urea (120 mg), and SDS (120 mg) were added into a Teflon-lined autoclave containing 16.7 mL water to form a suspension. After a few minutes stirring, the laser fabricated Cu foam was immersed into the suspension, which was latter placed into a homogeneous reactor at 120°C for a different amount of time (2, 4, 6, 8, and 10 h). Eventually, the products were cooled in air and washed repeatedly with ethanol, followed by drying in vacuum. The mass loading of Co(OH)_2_ catalysts on the laser ablated Cu foam (Cu/Cu Oxides/Co(OH)_2_-8 h) is estimated based on EDS results, i.e., 10.21 mg/cm^2^.

### Materials Characterizations

X-ray diffraction (XRD) was performed on a Mini Flex 600 using Cu Kα radiation (λ = 0.1542 nm) with a scan rate of 2° min^−1^. The morphologies of the samples were recorded by a Mira3 scanning electron microscope (SEM) and transmission electron microscope (TEM) images were obtained on Tecnai G2 F20 instruments operating at an accelerating voltage of 200 kV. The elemental composition and the valence states of the samples were detected by X-ray photoelectron spectroscopy (XPS) on SPECS ultrahigh vacuum system (Liu et al., 2015, 2019; Xie et al., 2016).

### Electrochemical Measurements

All electrochemical measurements were carried out with a Chenhua CHI 760E electrochemical workstation using a standard three-electrode system in 1 M KOH solution (pH 13.8) at room temperature. The prepared samples, a graphite rod, and a Hg/HgO electrode were used as the working electrode, counter electrode, and reference electrode, respectively. The liner sweep voltammograms (LSVs) were measured at a scan rate of 5 mV s^−1^ with iR compensations, where the Tafel slope can be derived from. The electrochemical impedance spectroscopy (EIS) measurement was performed at an electrolysis potential of 1.5 V vs. RHE over a frequency range from 0.01 to 100 kHz with ac amplitude of 5 mV. A simplified Randles equivalent circuit was used to simulate the Nyquist plot. The capacitance (normalized by the area) was determined from the cyclic voltammetry (CV) scans. The CV scans with various scan rates (5, 10, 20, 40, 80, 120, 160, and 200 mV s^−1^) were recorded in a non-faradaic potential range (0–0.1 V vs. Hg/HgO). The full water splitting measurements is carried out with a membrane to separate the H_2_ and O_2_, where the LSV curves are not iR corrected. The distance between the two electrodes is about 5 cm. Faradaic efficiency was measured toward gas chromatography experiment (GC-2014C, SHIMADZU) and the Faradaic efficiency of Cu/Cu Oxides/Co(OH)_2_-8 h is about 98.4%. All potentials measured in this work were converted to reversible hydrogen electrode (RHE) scale using the Nernst equation: *E*_*RHE*_ = *E*_*Hg*/*HgO*_ + 0.059 × *pH* + 0.098. The overpotential is estimated by *E*_*RHE*_ − 1.23.

## Results and Discussions

**Figure 1:**
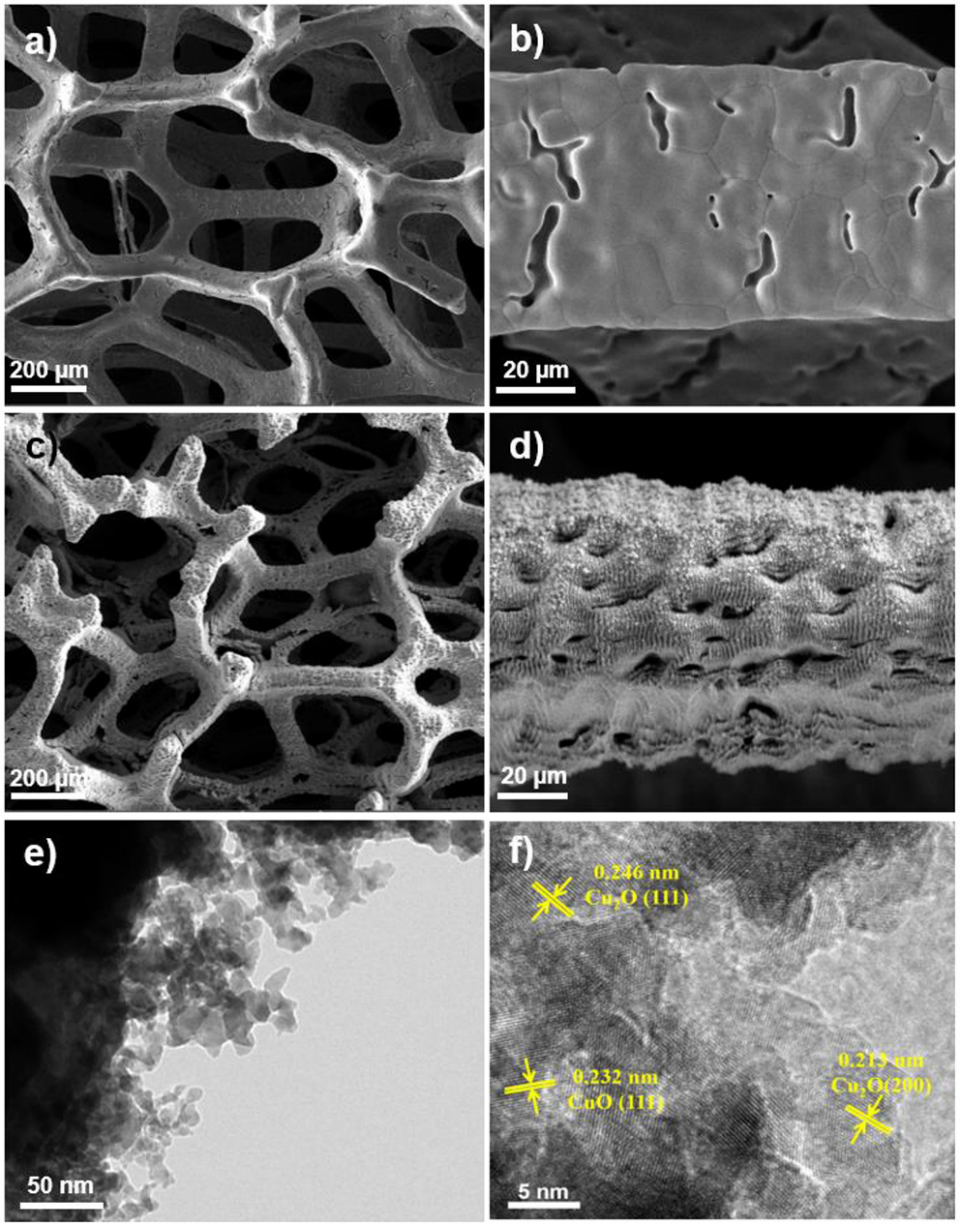
**(a,b)** SEM images of pristine Cu foam. **(c,d)** SEM images of the laser fabricated Cu foam. **(e,f)** TEM and HRTEM images of the laser fabricated Cu foam.

The morphology evolution from the pristine Cu foam, over laser ablation, to precipitation conversion was demonstrated by SEM and TEM characterizations. As shown in Figure 1, the smooth Cu surface turns to be with rich micro/nano-structures after laser ablation, which are composed of nanoparticles with a size of a few to a few tens of nanometers (Figure 1e). The HRTEM images of the laser fabricated Cu foam show lattice spacing of 0.246, 0.213, and 0.232 nm, corresponding to Cu_2_O (111), Cu_2_O (200), and CuO (111), respectively (Figure 1f), which suggests the oxidization of the Cu surface after laser ablation. These micro/nano-structures significantly increase the surface area with rich defects and superhydrophilicity (Li et al., 2019a), which can be beneficial to the growth of the electrocatalysts and increases the active sites as well as the catalytic activity. To compare the specific surface area of the Cu foam before and after the laser ablation, we conducted Brunauer-Emmett-Teller (BET) measurements of the fabricated samples. It shows that the BET specific surface area of pristine Cu foam is 0.22 m^2^/g, while that of laser ablated Cu foam increases to 0.98 m^2^/g, indicating the formation of micro/nano-structures. Figure S1 presents the water wettability of the pristine and the laser ablated Cu foam, which confirms the superhydrophilicity of the laser ablated Cu foam.

**Figure 2:**
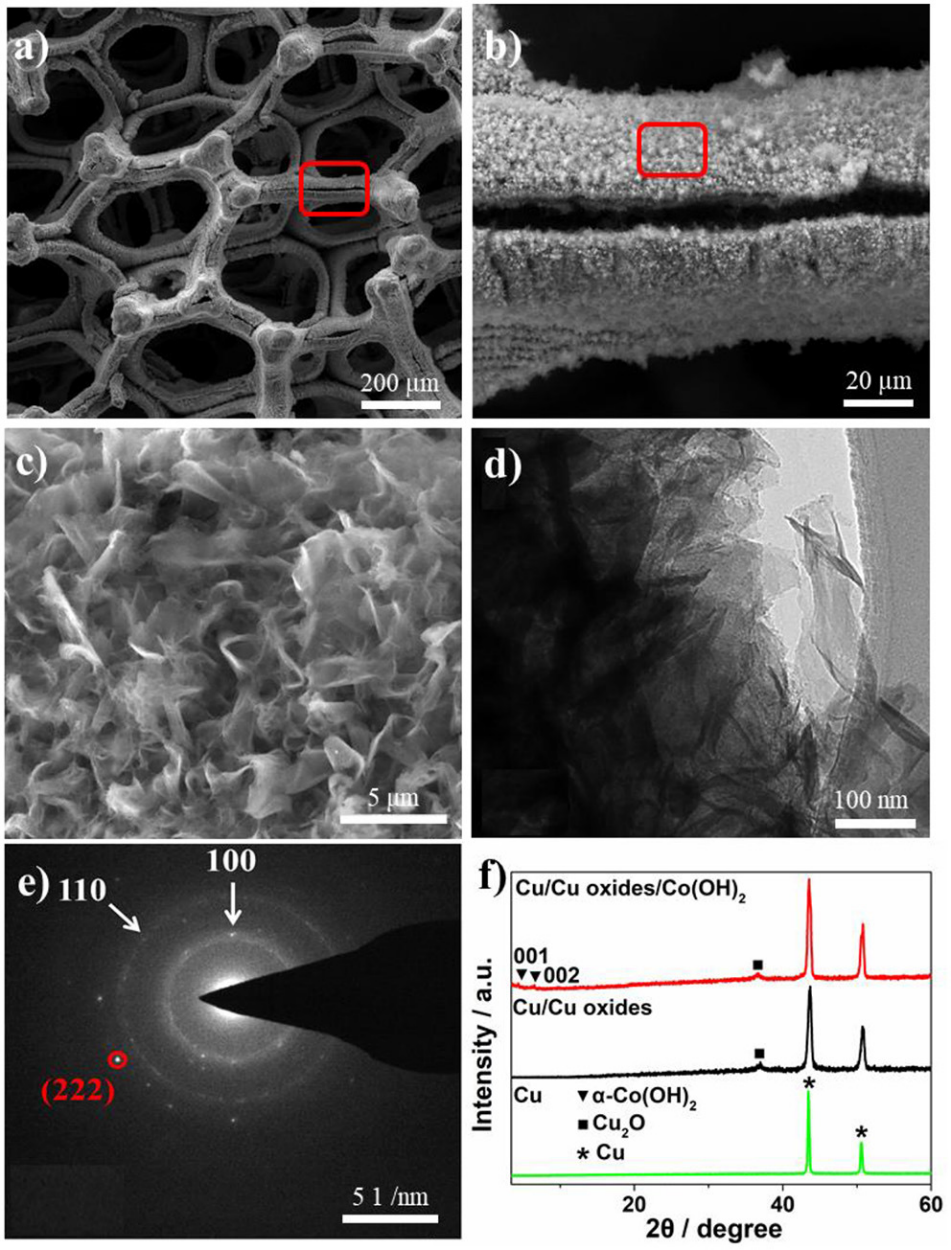
**(a–c)** SEM images of Cu/Cu oxides/Co(OH)_2_-8 h sample with different magnifications. **(d)** TEM and **(e)** SAED pattern images of Cu/Cu oxides/Co(OH)_2_-8 h. **(f)** XRD of the pristine Cu foam, laser fabricated Cu foam, and Cu/Cu oxides/Co(OH)_2_-8 h.

After the growth of Co(OH)_2_, clear layered Co(OH)_2_ nanosheets were found on the laser fabricated Cu surface with a lateral dimension of a few hundreds of nanometers to a few micrometers (Figures 2a–d). The two ordered diffraction rings of the selected area electron diffraction (SAED) pattern can be assigned to (100) and (110) of Co(OH)_2_ (Figure 2e; Liu et al., 2014), where one can also see diffraction patterns of Cu_2_O, i.e., (222). Figure 2f shows the X-ray diffraction pattern of different samples. The pristine Cu foam only contains the characteristic peaks of bare Cu, while the laser ablation induced the formation of Cu oxides, corresponding to the (111) of Cu_2_O (JCPDS 78-2076). No clear CuO peak can be found in the XRD pattern, probably due to the low content in the samples. After the precipitation conversion, two small peaks appear around 4.5° and 6.5°, which can be assigned to the (001) and (002) planes of α-Co(OH)_2_, respectively (Liu et al., 2014). The existence of Cu, Co, and O elements is further confirmed by the energy dispersive X-ray (EDX) elemental mapping images (Figure S2).

**Figure 3:**
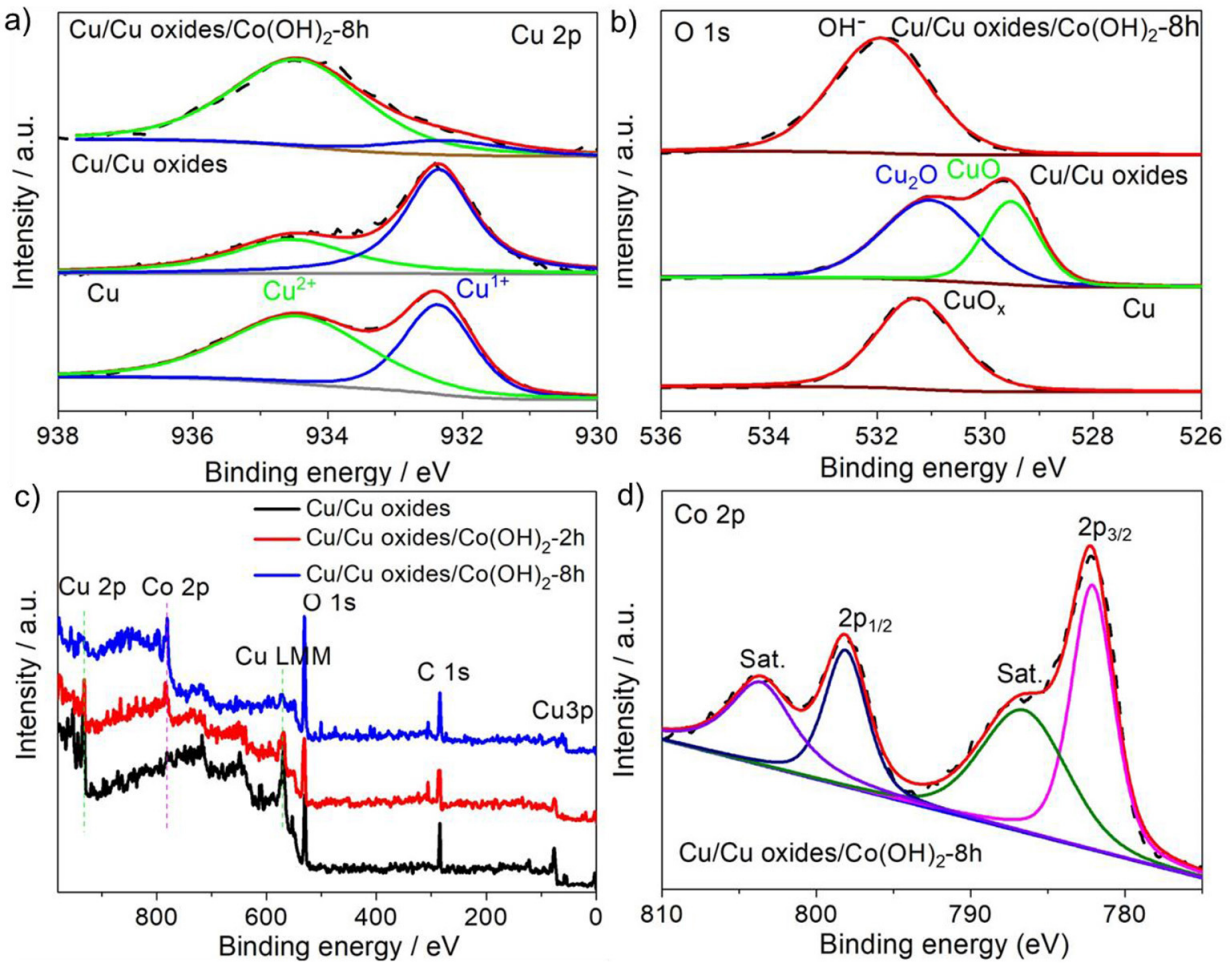
High-resolution XPS spectra of **(a)** Cu 2p and **(b)** O 1s of different samples (the pristine Cu foam, laser fabricated Cu foam, and Cu/Cu oxides/Co(OH)_2_-8 h). **(c)** Full XPS spectra of the laser fabricated Cu foam, Cu/Cu oxides/Co(OH)_2_-2 h, and Cu/Cu oxides/Co(OH)_2_-8 h. **(d)** High-resolution XPS spectra of Co 2p for Cu/Cu oxides/Co(OH)_2_-8 h. The dash lines indicate the pristine XPS spectra, while the colorful solid lines correspond to the fitted data.

XPS measurements were performed to identify the chemical valence states of the different elements in the samples. The pristine Cu foam shows slight surface oxidation, while the laser ablation not only creates rich micro/nano-structures but also accelerates the oxidation with a sharp increase of the Cu^1+^ peak (Figure 3a) and a broadened O 1s peak (Figure 3b). After the precipitation conversion, the abrupt increase of Cu^2+^ peak indicates a further oxidation of surface Cu_2_O to CuO during the process. The successful growth of Co(OH)_2_ is verified by the appearance of OH^−^ in the O 1s spectra and Co 2p peak, and the gradual disappearance of the Cu peak (Figures 3b,c). The Co 2p peak splits into 2p 1/2 and 2p 3/2 located at 798.0 and 782.1 eV (Figure 3d), with a spin orbital splitting energy of about 16 eV, in agreement with the binding energies for the Co^2+^ oxidation state (Jiang et al., 2016). Moreover, the shake-up peak of Co 2p_3/2_ further confirms the existence of Co^2+^.

**Figure 4:**
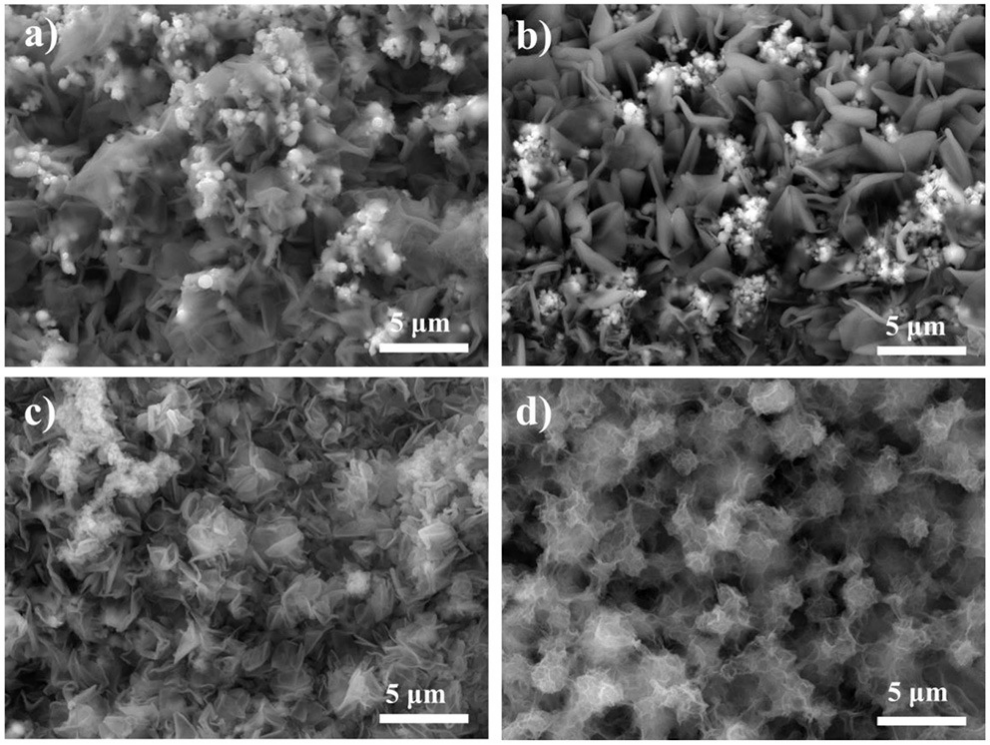
SEM images of the synthesized samples with different reaction time in the reactor **(a)** 2 h, **(b)** 4 h, **(c)** 6 h, and **(d)** 10 h.

Since the catalytic activity of the electrocatalysts depends on the morphology, the laser fabricated Cu foams were placed in the reactor with different reaction time (2, 4, 6, 8, and 10 h) under the same condition, in order to explore the reaction time dependent morphology evolution, which is characterized by SEM (Figure 4). In general, the amount of Co(OH)_2_ increases with the increasing of reaction time (See more SEM images with different magnifications in Figures S3–S6). For Cu/Cu oxides/Co(OH)_2_-2 h, the laser fabricated Cu foam was partially coated with Co(OH)_2_ nanosheets, where one can still see Cu oxide micro-structures. Increasing the reaction time up to 8 h (Figure 2c), the sample is completely covered by Co(OH)_2_ nanosheets from the SEM images. Further increasing the reaction time only results in the overgrowth of Co(OH)_2_ on the surface with clear agglomeration. This is consistent with the XPS results. As shown in Figure 3c, with the increasing of preparation time, the Co 2p peak increases, while the Cu 2p and Cu LMM peaks decrease, indicating the gradual growth of Co(OH)_2_ on the foams.

**Figure 5:**
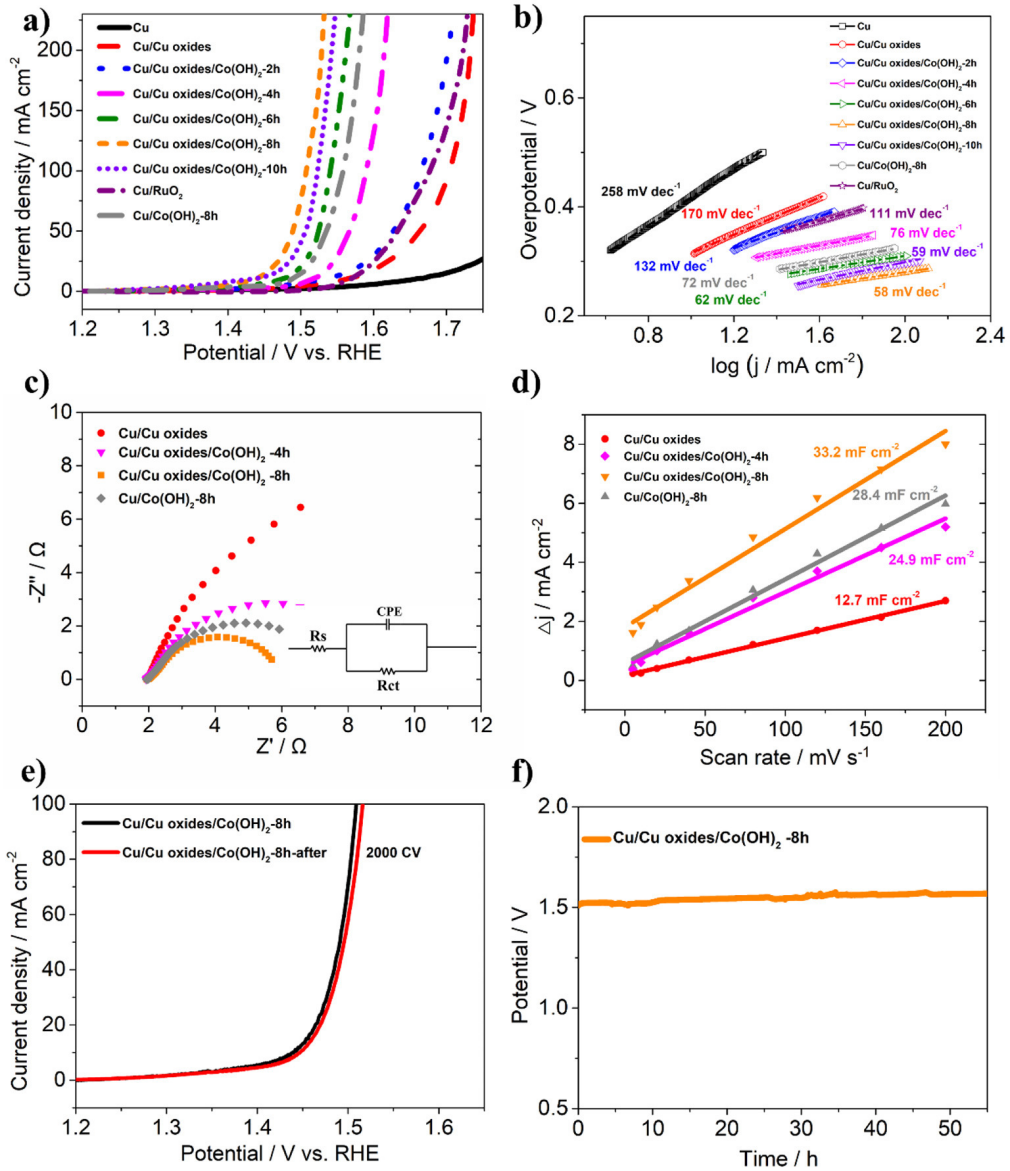
**(a,b)** LSV curves and Tafel plots of the synthesized samples for OER, respectively. **(c)** Nyquist plots of different samples at an applied potential of 1.50 V vs. RHE. Inset: the electrical equivalent circuit model for the Nyquist plot. **(d)** Capacitive currents at 0.96 V as a function of scan rates. **(e)** LSV of Cu/Cu Oxides/Co(OH)_2_-8 h before and after 2,000 CV segments. **(f)** Chronopotentiometry curve of Cu/Cu oxides/Co(OH)_2_-8 h at 10 mV cm^−2^.

To further evaluate the OER performance, the prepared samples were tested in 1 M KOH using a standard three-electrode setup. From the LSV polarization curves (Figure 5a), it is shown that the laser ablated Cu foams possess certain OER activity due to the fabricated rich micro/nano-structures, compared to the pristine Cu foams. After the growth of Co(OH)_2_, the synthesized samples show clearly enhanced OER activity. In consistent with the morphology evolution as demonstrated in Figure 4, the OER activity of the samples rises with the increasing of the reaction time, while the overgrowth of Co(OH)_2_ only results in a declined activity due to the decreased active sites as well as an increased resistance (Jiang et al., 2016). Among them, Cu/Cu oxides/Co(OH)_2_-8 h exhibits the best OER activity, with an overpotential of only 259 mV at 50 mA cm^−2^ and a Tafel slope of 58 mV dec^−1^ (Figures 5a,b), much smaller than those of the other samples. This activity is comparable or superior to the similar OER electrocatalysts reported in literature (Table S1; Jiang et al., 2016; Liu et al., 2016; Han et al., 2017; Huan et al., 2017; Zhang et al., 2017; Czioska et al., 2018; Li et al., 2018). For comparison, Co(OH)_2_ nanosheets are also grown on pristine Cu foam (Cu/Co(OH)_2_-8 h) with an overpotential of 305 mV at 50 mA cm^−2^ and Tafel slope of 72 mV dec^−1^, much larger than these of Cu/Cu oxides/Co(OH)_2_-8 h. Moreover, the effect of laser speed on the OER performance of the samples (Figure S7) is also investigated, where it is obvious that the 3 m/s sample shows better catalytic performance. It can be due to the more micro/nano-structures induced during the slow laser ablation process, which thus increases the growth sites for Co(OH)_2_ as well as the active sites for the reactivity. However, one should also note that the Cu foams can be damaged if the scanning speed is too slow. To compare with, commercial RuO_2_ shows an overpotential of 400 mV at 50 mA cm^−2^ and Tafel slope of 111 mV dec^−1^, also much larger than these of Cu/Cu oxides/Co(OH)_2_-8 h. According to the reaction pathway proposed by Krasil'shchikov (Doyle and Lyons, 2016), the Cu/Cu oxides/Co(OH)_2_-8 h have a Tafel slope of 58 mV dec^−1^, indicating that the second electron transfer process, i.e., *MO*^−^ → *MO* + *e*^−^, maybe the rate-determining step (RDS) of OER generation. To further understand the catalytic performance, electrochemical impedance spectroscopy was carried out (Figure 5c) and the fitted results of Nyquist plot are shown in Table S2, where Cu/Cu oxides/Co(OH)_2_-8 h has the smallest semicircle radius, indicating faster catalytic kinetics. Figure 5d depict the capacitance (normalized by the area) estimated by cyclic voltammetry (CV) curves measured at different scan rates for different samples (Figure S8). It is clearly shown that the capacitance of Cu/Cu oxides/Co(OH)_2_-8 h (33.2 mF cm^−2^) is larger than that of Cu/Cu oxides (12.7 mF cm^−2^), Cu/Cu oxides/Co(OH)_2_-4 h (24.9 mF cm^−2^), and Cu/Co(OH)_2_-8 h (28.4 mF cm^−2^) due to the growth of Co(OH)_2_ and the increased active sites from laser ablation.

To evaluate the mild-term durability of the prepared electrocatalysts, the LSV after 2,000 CV of Cu/Cu oxides/Co(OH)_2_-8 h was tested. As shown in Figure 5e, the overpotential only shows an increase of 5 mV at 50 mA cm^−2^ after the test. Chronopotentiometry test was carried out to further confirm the good long-term stability (Figure 5f), where the potential of Cu/Cu oxides/Co(OH)_2_-8 h does not show obvious increase after about 55 h at 10 mA cm^−2^. In addition, both TEM and SEM images demonstrate that the morphology of the samples retains without significant changes after reaction (Figures S9, S10), confirming its excellent mild-term stability. The Faradaic efficiency (FE) of Cu/Cu Oxides/Co(OH)_2_-8 h was carried out together with the gas chromatography experiment (see details in Figure S11). We conduct the experiment with two voltages (1.51 and 1.55 V vs. RHE), where the corresponding FE are estimated to be 96.5 and 98.4%.

**Figure 6:**
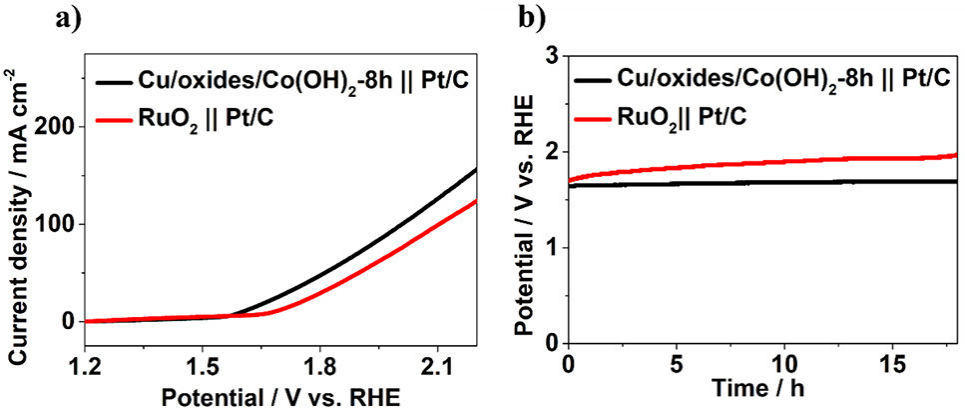
**(a)** LSV curves for overall water splitting without iR compensation. **(b)** Chronopotentiometry durability test at a constant current density of 10 mA cm^−2^.

To further evaluate the possibility of Cu/Cu oxides/Co(OH)_2_ for practical utilization, a water splitting system was built with Cu/Cu oxides/Co(OH)_2_-8 h and commercial Pt/C as anode and cathode, respectively. In comparison with the commercial RuO_2_ || Pt/C (Figure 6), the Cu/Cu oxides/Co(OH)_2_-8 h || Pt/C system displays a lower overpotential to reach 10 mA cm^−2^ (371 mV compared to 451 mV). For chronopotentiometry durability test, the RuO_2_ || Pt/C system shows a clear increase of potential with time, while that of the Cu/Cu oxides/Co(OH)_2_-8 h || Pt/C system stays up to 18 h, indicating better mild-term durability. In general, the enhanced OER activity of the laser fabricated samples can be attributed to the following reasons: (1) The produced Cu oxides have certain OER activity, compared to bare Cu foam. (2) The laser fabricated Cu surfaces with rich micro/nano-structures and large surface area are helpful for the growth of Co(OH)_2_ nanosheets, thus with more active sites. (3) The fabricated micro/nano-structures possess certain porosity and are superhydrophilic, which can be conducive to the diffusion of water molecules and to the release of gas molecules thus fast OER kinetics for the prepared catalysts.

## Conclusions

In summary, a highly active OER electrocatalyst, i.e., Cu/Cu oxides/Co(OH)_2_, has been successfully synthesized through a combination of laser fabrication and precipitation conversion method. It only requires a low overpotential of 259 mV to reach a current density of 50 mA cm^−2^ in 1 M KOH, associated with superior durability of more than 55 h and a small Tafel slope of 58 mV dec^−1^, due to significantly improved active sites and the superhydrophilicity from the laser fabrication. Our study provides a new strategy to the design and construction of high efficient non-noble metal based OER catalysts.

## Data Availability Statement

All datasets generated for this study are included in the article/Supplementary Material.

## Author Contributions

XZ carried out the experiments and data analysis, as well as wrote the manuscript. KY modified the substrates with laser ablation. WQ and YL co-designed the experiments. WQ, NZ, SG, ZL, and YL discussed the experimental data and revised the manuscript together.

### Conflict of Interest

The authors declare that the research was conducted in the absence of any commercial or financial relationships that could be construed as a potential conflict of interest.

## Supplementary Material

The supporting information includes: EDS mapping images of the synthesized Cu/Cu oxides/Co(OH)_2_ electrocatalysts, SEM images and CVs of the synthesized catalysts, TEM and SEM images of the Cu/Cu Oxides/Co(OH)_2_-8 h after reaction, detailed information of gas chromatography experiment, the fitted results of the Nyquist plot, comparison of OER performances with literature results.
