# Supplementary material for: Co(OH)2 Nanosheets Supported on Laser Ablated Cu Foam: An Efficient Oxygen Evolution Reaction Electrocatalyst
Source: Front Chem. 2020 Jan 10;7:900. doi: 10.3389/fchem.2019.00900 (PMC6966496; doi:10.3389/fchem.2019.00900)
Supplement: Supplementary file 1 [file Data_Sheet_1.PDF]

Supporting Information

$Co(OH)_2$ nanosheets supported on laser fabricated Cu foam: An Efficient Oxygen Evolution Reaction Electrocatalyst

Xinfeng Zhou, [1] Weihong Qi, [1,2] * Kai Yin, [3] Ning Zhang, [1] Shen Gong, [1] Zhou Li, [1] Yejun Li [1,3]*

[1] School of Materials Science and Engineering, Central South University, Changsha 410083, P. R. China

[2] State Key Laboratory of Solidification Processing, Center of Advanced Lubrication and Seal Materials, Northwestern Polytechnical University, Xi'an, Shanxi 710072, P. R. China

[3] Hunan Key Laboratory of Super Microstructure and Ultrafast Process, School of Physics and Electronics, Central South University, Changsha 410083, P. R. China

*Corresponding author. E-mail: qiwh216@nwpu.edu.cn; yejunli@csu.edu.cn

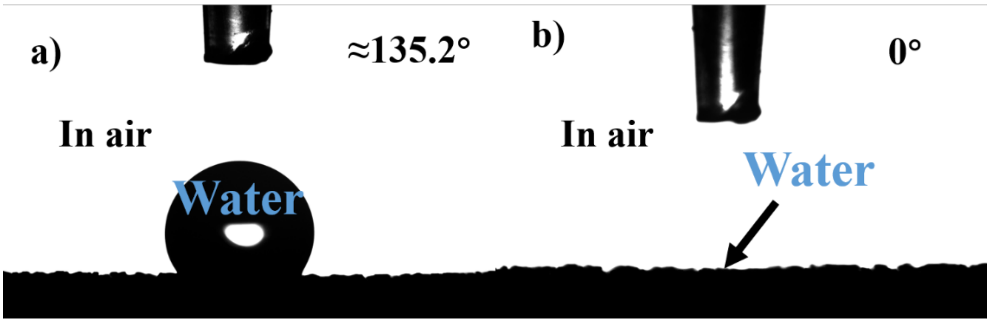

Figure S1. Water wettability of (a) pristine Cu foam and (b) the laser ablated Cu foam in air, respectively.

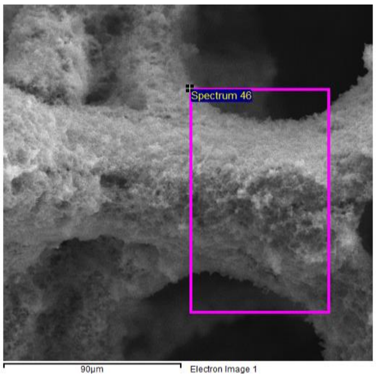

Element	Weight%	Atomic%	
O K	30.41	62.31	
S K	2.03	2.08	
Co K	18.87	10.50	
Cu K	48.68	25.12	
Totals	100.00		

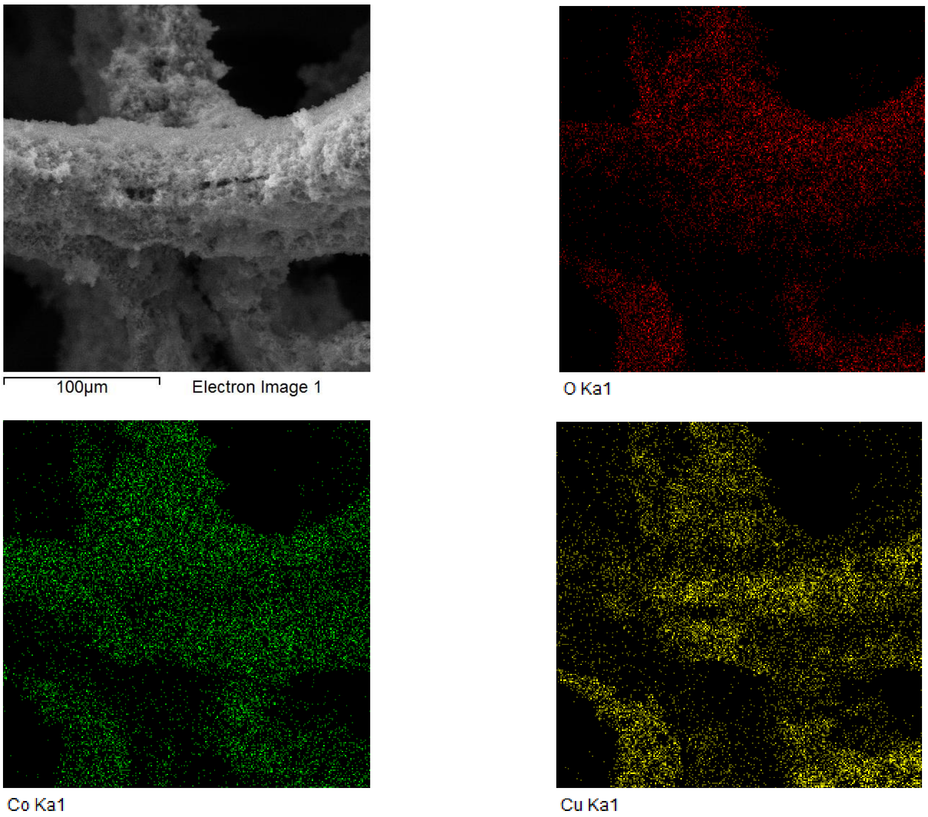

Figure S2. EDS mapping images of the synthesized Cu/Cu oxides/$Co(OH)_2$ electrocatalysts.

The existence of a small amount of element S could be attributed to the residual of SDS in the products.

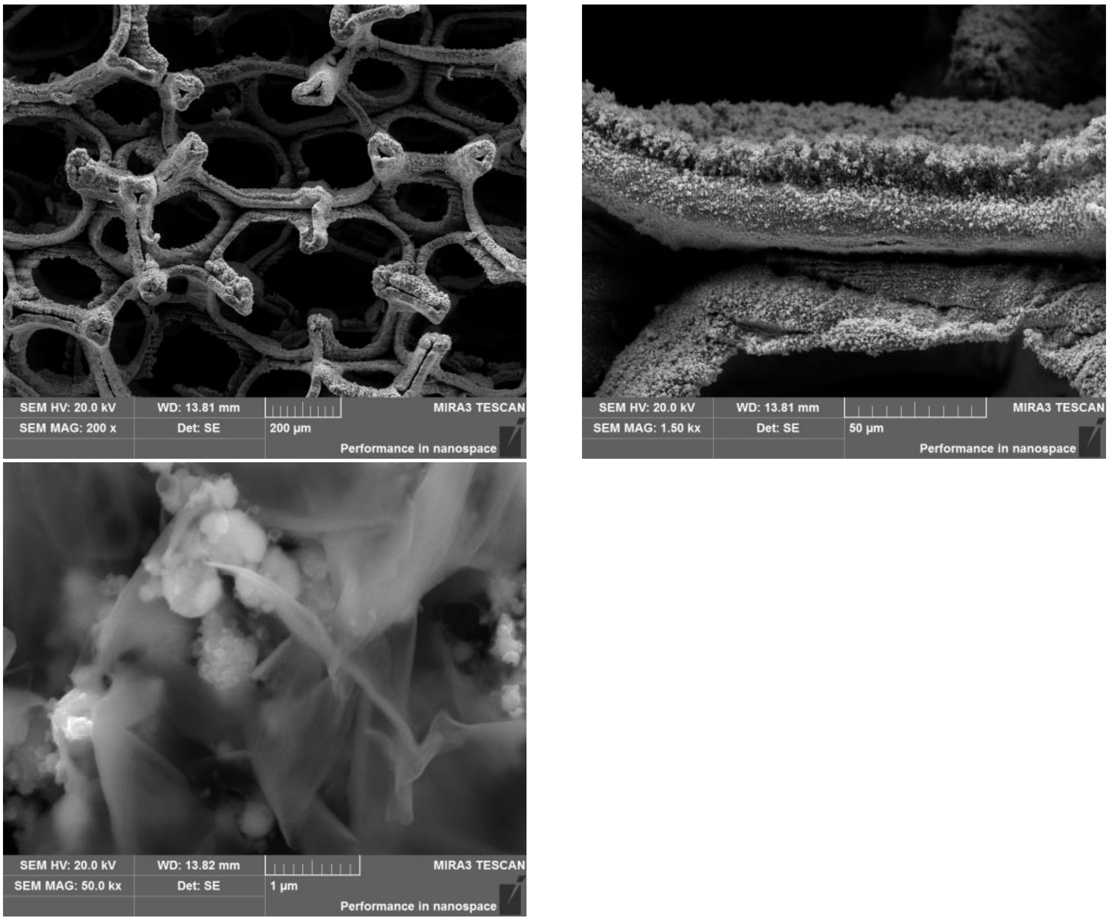

Figure S3. SEM of Cu/Cu oxides/$Co(OH)_2$-2h.

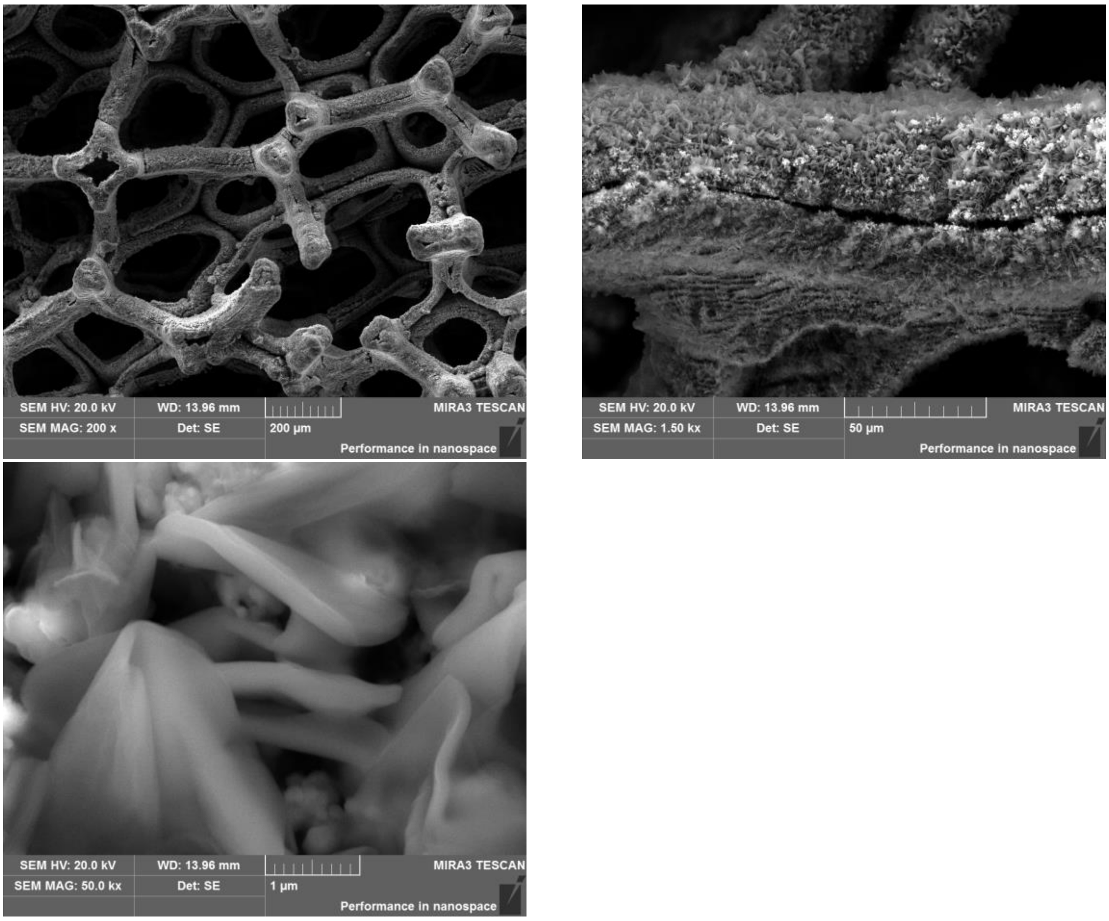

Figure S4. SEM of Cu/Cu oxides/$Co(OH)_2$-4h.

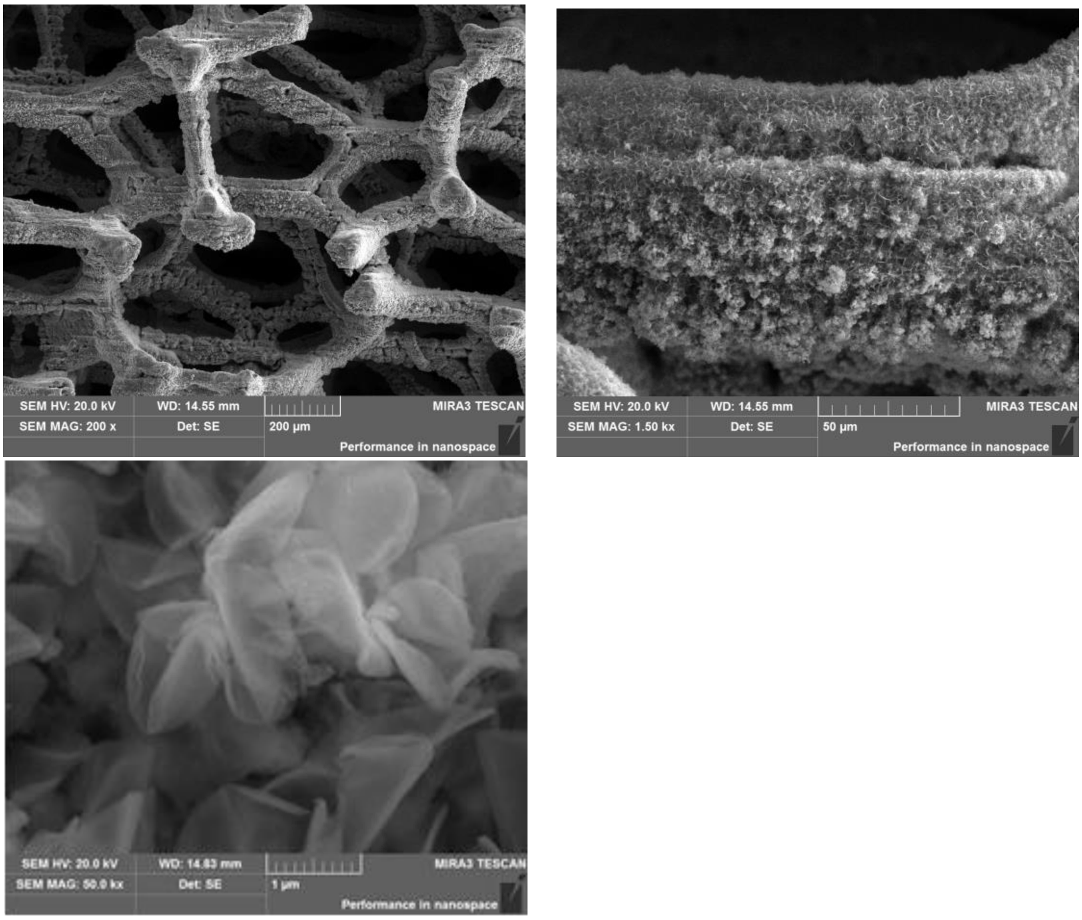

Figure S5. SEM of Cu/Cu oxides/$Co(OH)_2$-6h.

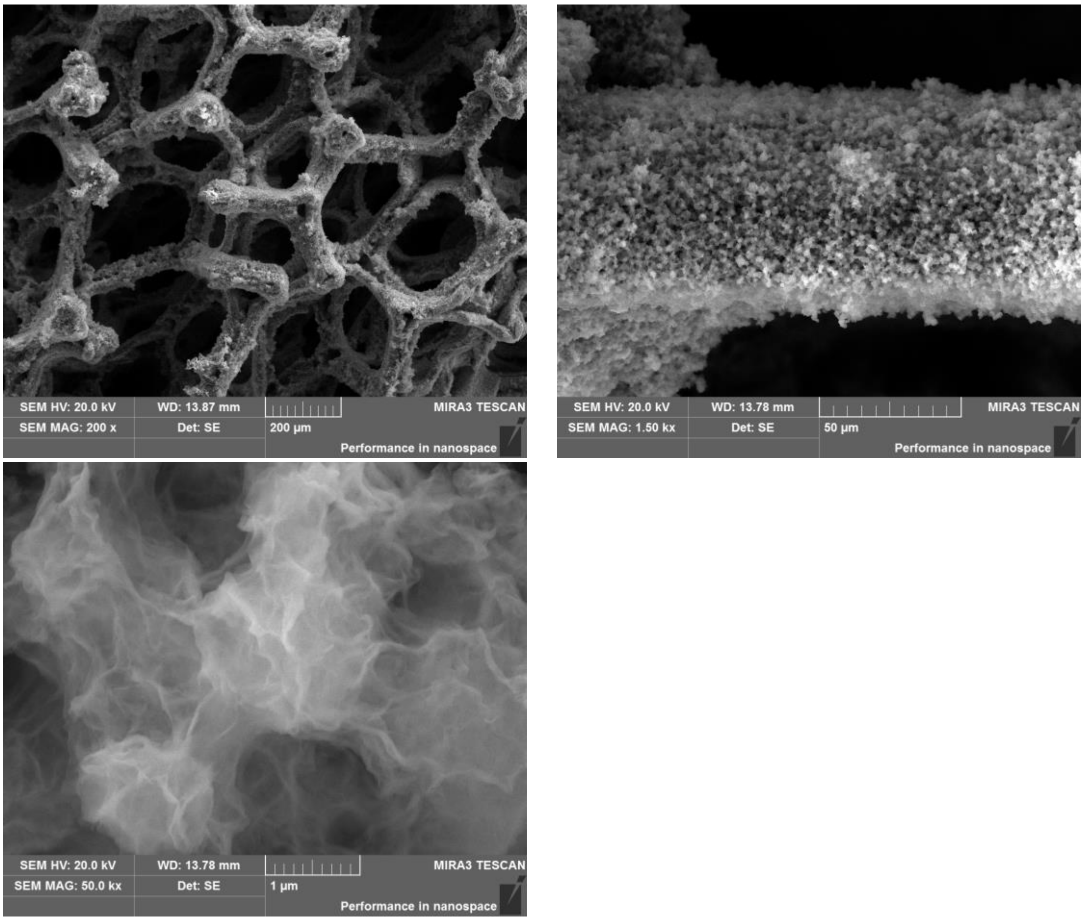

Figure S6. SEM of Cu/Cu oxides/$Co(OH)_2$**-**10h.

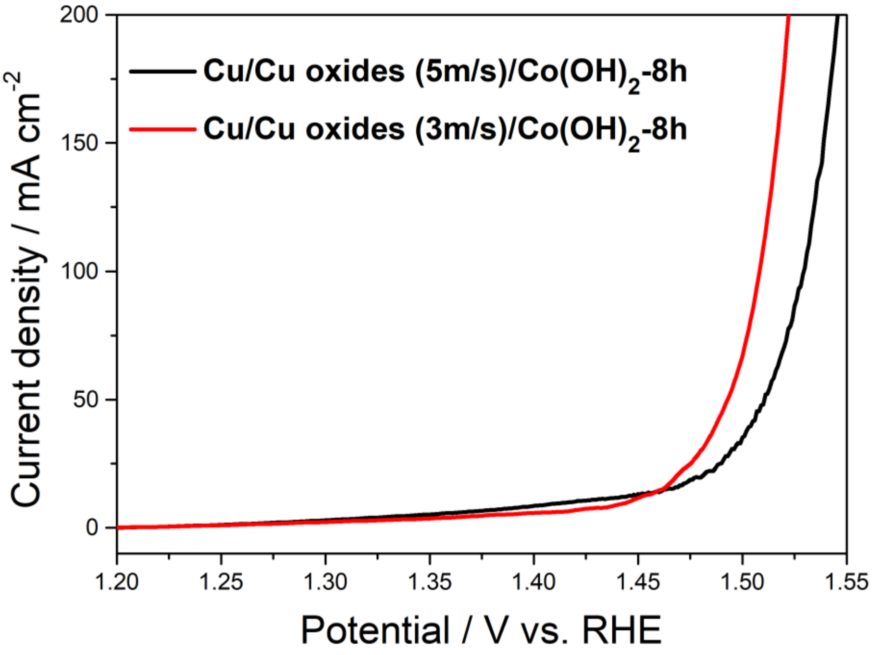

Figure S7: LSV of the Cu/Cu oxides (5m/s) /$Co(OH)_2$-8h and Cu/Cu oxides (3m/s) /$Co(OH)_2$-8h.

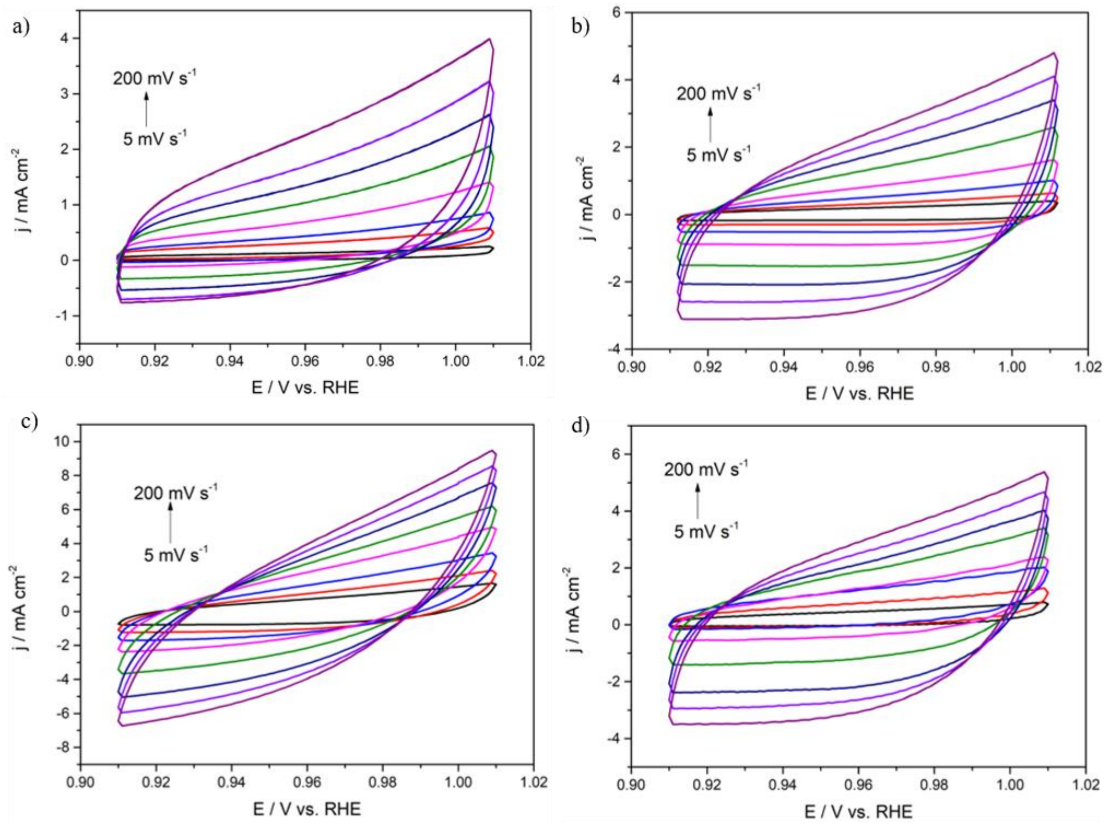

Figure S8. CVs of (a) laser fabricated Cu foam, (b) Cu/Cu oxides/$Co(OH)_2$-4h, (c) Cu/Cu oxides/$Co(OH)_2$-8h, and (d) Cu/$Co(OH)_2$-8h at scan rates of 5, 10, 20, 40, 80, 120, 160, and 200 mV s^{-1}.

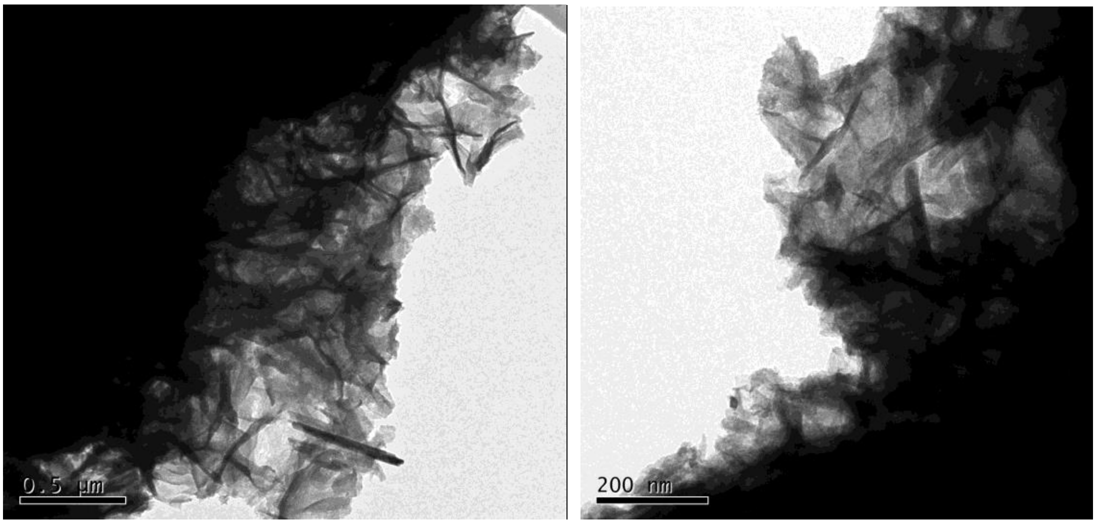

Figure S9. TEM images of sample Cu/Cu oxides/Co(OH)$_2$-8h after reaction.

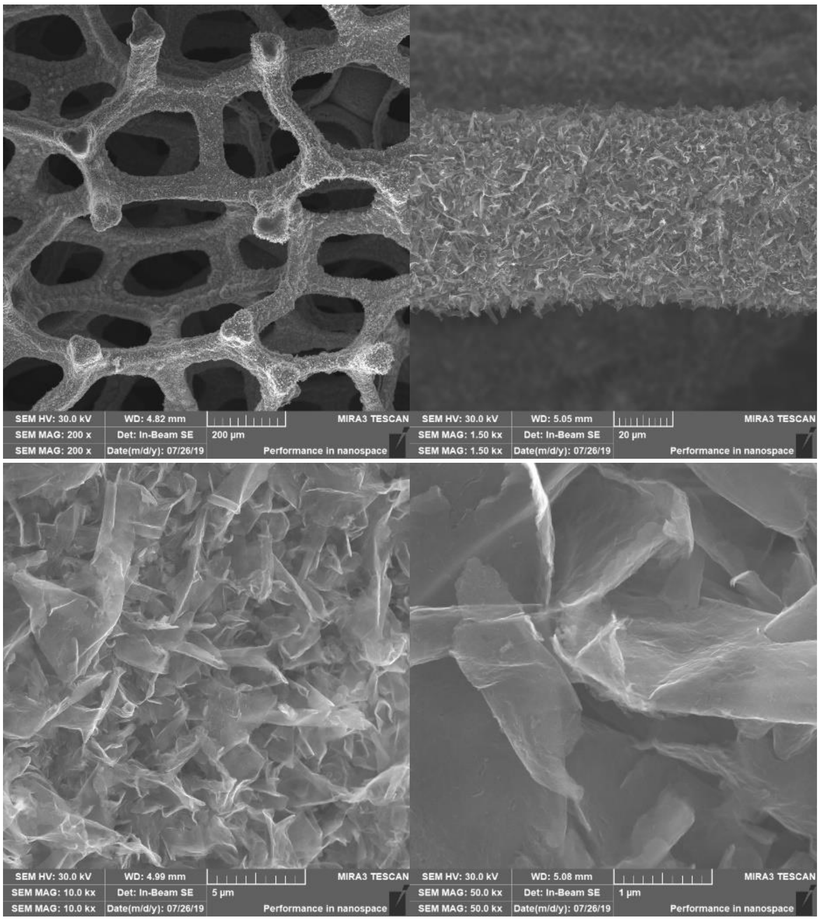

Figure S10. SEM images of Cu/Cu Oxides/Co(OH)$_2$-8h after reaction

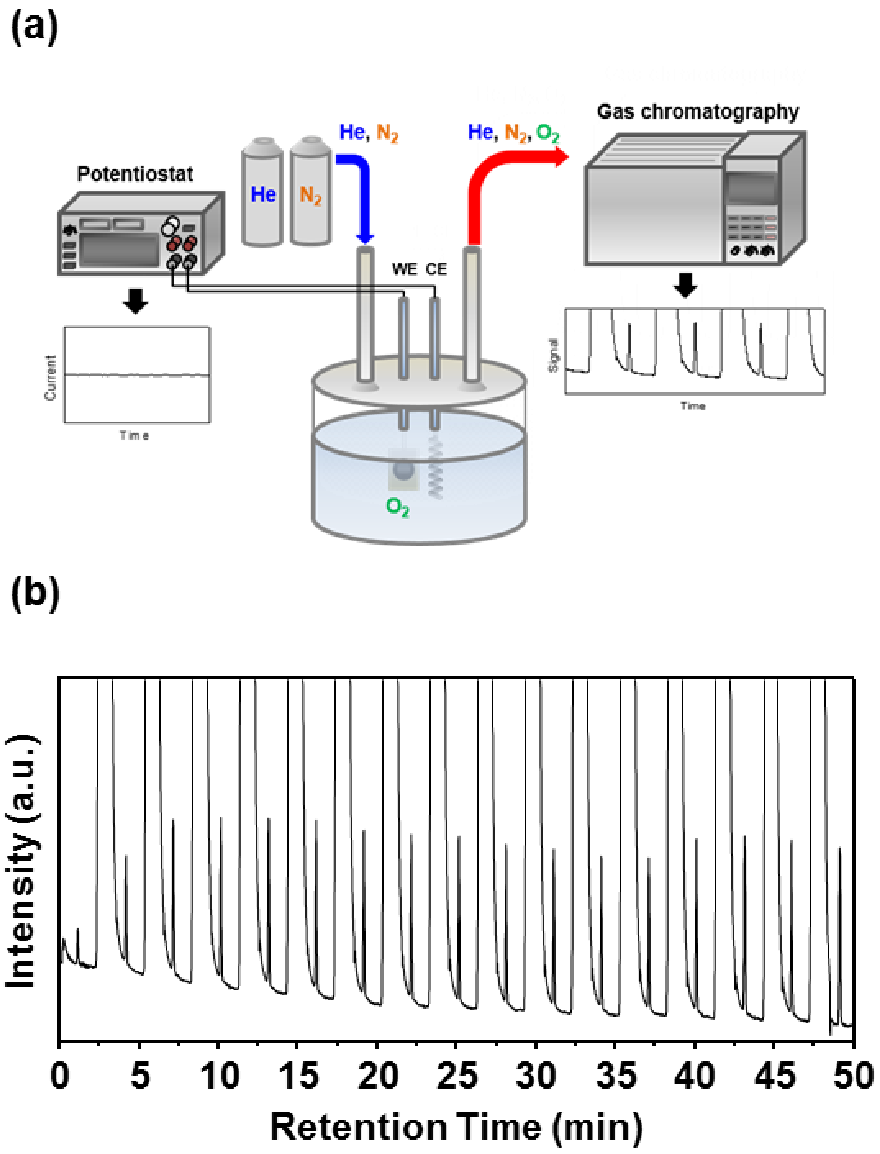

Figure S11 (a) Schematic diagram of gas chromatography experiment and (b) raw GC response of Cu/Cu Oxides/Co(OH)2-8h with respect to retention time.

Scheme of gas chromatography experiment was suggested in Fig. R11. Experimental details were established referring to earlier gas experiment reports [12,13]. Chromatogram of GC analysis (Fig. S19) indicates evolution of oxygen peak in every 3 min. Since current can roughly consider as constant numerical value (Voltage keep

constant), oxygen generation assuming 100% Faradaic efficiency may be calculated. Practically measured oxygen is estimated with conversion of oxygen peak area to volumetric fraction of oxygen, using 20.95% of oxygen ratio of fresh air as a reference. As shown in Fig. S9b, oxygen peak emerges as the time increases, then becomes saturated after 6 min of operation. Smaller oxygen peak at earlier stage (< 6 min) is because surface was being oxidized during OER and steady-state had still not achieved in short time period. Oxygen peak intensity attains maximum as the system reaches steady-state, which means that generation rate on the electrode becomes equivalent to output rate.

Amount of measured gas was calculated with accumulating generated oxygen by assuming constant generation rate during each 3 min period. Ideal O_2 production (assumed 100% Faradaic efficiency) was calculated with following relation:

$$\mathrm{i\ mA} = \mathrm{i}\frac{\mathrm{mC}}{s} = \frac{i * 10^{-3}\ C/s}{96485\ C/eq \times 4\ eq/mol} = i * 2.591 \times 10^{-8}\ mol/s$$

Volumetric fraction of evolved oxygen is then calculated with ideal gas law, then compared to standard peak (oxygen peak of fresh air). Faradaic efficiency was calculated with dividing measured generation rate by theoretical generation rate.

$$\text{Faraidac Efficiency (FE)} = \frac{Measured\ (actual)\ generation}{Theoretical\ (calculated)\ generation} \times 100\%$$

We conduct the experiment with two Voltage (1.51V, 1.55V Vs RHE), the corresponding current are 17.86 mA, 46.5 mA, the corresponding Faraidac Efficiency (FE) are 96.5%, 98.4%.

Table 1. The fitted results of the Nyquist plot.

Samples	Rs	Rct	CPE-T	CPE-P
Cu/Cu Oxides -4h	1.598	8.331	0.50914	0.78036
Cu/Cu Oxides -8h	1.424	4.52	0.89033	0.78446
Cu/Cu Oxides	1.427	51.89	0.62501	0.72799
Cu/Co(OH)2-8h	1.492	6.115	0.72217	0.76917

Table 2. Electrocatalytic Performance List of Low-Cost Metal-based-Integrated Electrodes for oxygen evolution reaction (OER):

Samples	Electrolyte	j (mA cm^{-2})	η (mV)	Tafel slope (mV dec^{-1})	Substrate	Ref.
Co-CuO NA/CF	1 M KOH	50	299	134	Cu foam	1
NiFe-LDH/CuO NRs/CF	1 M KOH	50	320	60	Cu foil	2
Cu-$Co(OH)_2$	1 M KOH	10	300	47	NA	3
Cu/Cu_2O/CuO	1 M NaOH	10	290	64	Cu foam	4
Annealed CuO	1 M KOH	10	430	61.4	NA	5
$NiFeO_x$/CuO	1 M KOH	100	300	36	Cu foil	6
α-Co(OH)2	1 M KOH	10	350	76	NA	7
β-Co(OH)2	1 M KOH	10	445	75	NA	8
NiFe-LDH@Ni Fe-Bi/CC	1 M KOH	50	294	96	carbon cloth	9
Co_3O_4 NS/NF	0.1 M KOH	10	190	103	Ni foam	10
Cu_3P@NF	1 M KOH	10	320	54	Ni foam	11
**Cu/Cu oxides/ $Co(OH)_2$-8h**	**1 M KOH**	**50**	**259**	**58**	**Cu foam**	**This study**
**Cu/Cu oxides/ $Co(OH)_2$-8h**	**1 M KOH**	**10**	**210**	**58**	**Cu foam**	**This study**

1 X. Xiong, C. You, Z. Liu, A. M. Asiri and X. Sun, ACS Sustain. Chem. Eng., 2018, 6, 2883–2887.

2 Q. Zhou, T.-T. Li, J. Qian, W. Xu, Y. Hu and Y.-Q. Zheng, ACS Appl. Energy Mater., 2018, 1, 1364–1373.

3 L. Chen, H. Zhang, L. Chen, X. Wei, J. Shi and M. He, J. Mater. Chem. A, 2017, 5, 22568–22575.

4 T. N. Huan, G. Rousse, S. Zanna, I. T. Lucas, X. Xu, N. Menguy, V. Mougel and M. Fontecave, Angew. Chemie - Int. Ed., 2017, 56, 4792–4796.

5 M. Oberst, C. Bertsch, A. Lahm, S. Wuerstlin and U. Holz, Comput. Aided Surg., 2006, 11, 87–91.

6 S. Czioska, J. Wang, S. Zuo, X. Teng and Z. Chen, ChemCatChem, 2018, 10, 1005–1011.

7 H. Jin, S. Mao, G. Zhan, F. Xu, X. Bao and Y. Wang, J. Mater. Chem. A, 2017, 5, 1078–1084.

8 P. F. Liu, S. Yang, L. R. Zheng, B. Zhang and H. G. Yang, J. Mater. Chem. A, 2016, 4, 9578–9584.

9 L. Zhang, R. Zhang, R. Ge, X. Ren, S. Hao, F. Xie, F. Qu, Z. Liu, G. Du, A. M. Asiri, B. Zheng and X. Sun, Chem. - A Eur. J., 2017, 23, 11499–11503.

10 A. Li, C. Wang, H. Zhang, Z. Zhao, J. Wang, M. Cheng, H. Zhao, J. Wang, M. Wu and J. Wang, Electrochim. Acta, 2018, 276, 153–161.

11 A. Han, H. Zhang, R. Yuan, H. Ji and P. Du, ACS Appl. Mater. Interfaces, 2017, 9, 2240–2248.

12 Y. Chen, C. W. Li, M. W. Kanan, J. Am. Chem. Soc. 134 (2012) 19969-19972.

13 W. Zhu, R. Michalsky, Ö. Metin, H. Lv, S. Guo, C. J. Wright, X. Sun, A. A. Peterson, S. Sun, J. Am. Chem. Soc. 135 (2013) 16833-16836.
